# A Rare Case of Multiple Anomalies of the Coronary Arteries

**DOI:** 10.7759/cureus.2796

**Published:** 2018-06-13

**Authors:** Alexandar Iliev, Georgi Kotov, Iva N Dimitrova

**Affiliations:** 1 Anatomy, Histology and Embryology, Medical University of Sofia, Sofia, BGR; 2 Cardiology, University Hospital St. Ekaterina, Medical University of Sofia, Sofia, BGR

**Keywords:** coronary artery, anomalies, coronary angiography

## Abstract

Coronary artery anomalies represent a rare phenomenon, which is observed in approximately 1% of the population. Although not a frequent anomaly, they may lead to complications during various procedures and can sometimes present with angina pectoris or myocardial infarction. Herein, we present a rare case of a patient with origin of the left main coronary artery from the right sinus of Valsalva combined with a superdominant right coronary artery due to a hypoplastic left circumflex artery. A 67-year-old male patient presented with symptoms of chest pain on exertion, which lasted for approximately 10 minutes and resolved after rest. Physical examination, auscultation, electrocardiogram, and transthoracic echocardiography revealed normal findings. Cardiac enzymes were within the reference ranges, while the levels of triglycerides and low density lipoprotein (LDL)-cholesterol were elevated. The patient was further evaluated through coronary angiography. It revealed the origin of the left coronary artery from the right sinus of Valsalva, with a markedly hypoplastic left circumflex artery and the presence of a ‘superdominant’ right coronary artery. Atherosclerotic lesions were not observed, and the symptoms were discussed to have been caused by the anomalous pattern of the coronary arteries and the hypoplastic left circumflex artery in particular. The most common symptom of a hypoplastic or absent left circumflex artery is chest pain on exertion which is explained by the ‘steal’ phenomenon – due to increased demand in the area normally supplied by the left circumflex artery, a transitory ischemia occurs in the basins supplied by the left anterior descending artery and the right coronary artery. These findings were similar to our case. Such variations, although mostly asymptomatic, can sometimes lead to serious cardiovascular conditions and should be considered by clinicians during the assessment of cardiac symptoms.

## Introduction

Coronary artery anomalies (CAAs) represent a rare phenomenon, which is observed in approximately 1% of the population, with an incidence ranging from 0.3% to 5.6% [1]. Although not frequently encountered, they may lead to complications during procedures and can sometimes present with angina pectoris or myocardial infarction [2]. Furthermore, they are the second most common reason for sudden cardiac death among young athletes [1, 3]. The most common CAA is the separate origin of the left circumflex artery (LCX) and the left anterior descending artery (LAD), with an incidence of 0.41% [1, 4]. According to Angelini, the origin of the left main coronary artery (LMCA) from the right sinus of Valsalva (RSV) is extremely rare, accounting for 0.09%-0.15% of CAA [5]. This anomaly is considered as potentially serious and could be associated with acute myocardial ischemia and sudden death [5]. The absence of the LCX is sometimes considered a normal variant, and it is associated with the so-called ‘superdominant’ right coronary artery (RCA), which ensures compensatory blood supply to areas of the heart normally supplied by the LCX [1, 3, 6]. The absence of the LCX has been considered as a benign anomaly [4]. A LAD arising from the RSV is also a very rare variation, reported in 0.03% of cases and associated with tetralogy of Fallot or ventricular septal defect [4, 7].

Herein, we present a rare case of a patient with origin of the LMCA from the RSV combined with a superdominant RCA due to a hypoplastic LCA.

## Case presentation

**Figure 1:**
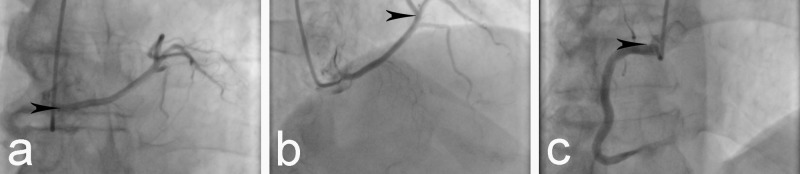
Coronary angiogram. a. Left main coronary artery taking off from the right sinus of Valsalva (black arrowhead). b. Left anterior descending artery taking off from the left main coronary artery in the usual way and providing septal branches (black arrowhead). c. Normal origin of the right coronary artery from the right sinus of Valsalva (black arrowhead).

**Figure 2:**
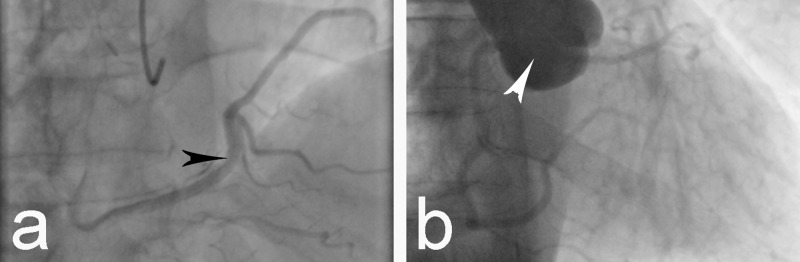
Coronary angiogram. a. Superdominant right coronary artery providing blood supply to the zone of the left circumflex artery (black arrowhead). b. Aortography showing origin of two coronary arteries from the right sinus of Valsalva and no vessels taking off from the left sinus of Valsalva (white arrowhead).

A 67-year-old male patient of Europid origin presented to the Clinic of Cardiology with symptoms of chest pain on exertion which lasted for approximately 10 minutes and resolved after rest. The patient reported that such events had been occurring for several years, but became more frequent over the previous three months. There was no history of syncope, but the patient reported cases of orthopnea with nocturnal dyspnea. Physical examination revealed a person with normosthenic build, arterial blood pressure 145/95 mm Hg, and pulse rate 65/min. Auscultation of the heart sounds was normal, without any pathological murmurs. The electrocardiogram showed a sinus rhythm and normal axis of the heart and revealed no data of ischemic damage or arrhythmias. Transthoracic echocardiography revealed normal ventricular systolic function with an ejection fraction (EF) of 60% and insignificant mitral regurgitation. Cardiac enzymes were within the reference ranges. Blood lipid tests revealed increased levels of triglycerides and low density lipoprotein (LDL)-cholesterol together with borderline low high-density lipoprotein (HDL)-cholesterol levels. The patient reported no history of tobacco use and claimed not to be exercising regularly. The suspected preliminary diagnosis was coronary artery disease, and so the patient was further evaluated through coronary angiography. It revealed the origin of the LMCA from the RSV, which then gave off the LCX and LAD (Figure 1a-1b). The LCX was found markedly hypoplastic. The RCA originated in the usual way from the RSV (Figure 1c), but was larger in size and was recognized as the so-called ‘superdominant’ RCA, which gave off branches for the territory normally supplied by the LCX (Figure 2a).

The left sinus of Valsalva (LSV) gave off no arteries (Figure 2b). Atherosclerotic lesions were not observed, and the symptoms were discussed to have been caused by the anomalous pattern of the coronary arteries.

## Discussion

In the present case we did not observe any branches taking off from the LSV. Instead, we noted a RCA and a LMCA both arising from the RSV. The LMCA can be classified into four major types depending on its course after taking off from the RSV: septal course (beneath the right ventricular infundibulum); anterior course (in front of the pulmonary artery); retro-aortic course (behind the aorta); and inter-arterial course (between the aorta and the pulmonary artery) [5]. The inter-arterial course is the least common but is considered the most malignant due to the fact that during physical activity, the LMCA can be compressed between the aorta and the pulmonary artery as a result of distension of these vessels [5, 8]. In the present case, the anomalous LMCA followed the anterior course and so the risk of compression was determined as insignificant, because of the higher systemic pressure compared to the low pressure in the pulmonary artery. Intussusception of the anomalous artery has also been proposed as a possible mechanism in the pathogenesis of acute myocardial ischemia and sudden death [8].

The absence of the LCX is most often a benign finding during coronary angiography but has been reported as symptomatic in approximately 20% of cases [3]. The most common symptom is chest pain on exertion which is explained by the ‘steal’ phenomenon – due to increased demand in the area normally supplied by the LCX, a transitory ischemia occurs in the basins supplied by the LAD and RCA [6]. These findings were similar to our case where the chest pain could not be explained by atherosclerosis and the zone of the hypoplastic LCX was supplied by branches from the RCA. These findings show that anomalies of the coronary arteries require prompt evaluation – and coronary angiography remains the reference standard for that [1].

## Conclusions

In conclusion, we have described a very rare multiple variation of the coronary arteries. Such variations, although mostly asymptomatic, can sometimes lead to serious cardiovascular conditions and should be considered by clinicians in the assessment of cardiac symptoms.
